# Chloral—A New Anæsthetic

**Published:** 1869-11

**Authors:** 


Chloral—A New Anæsthetic.—Monsieur Liebreich has presented a memoir to the Academie des Sciences, which contains some interesting details concerning a new anæsthetic he has just discovered. An important difference between this new chemical compound, which he calls "chloral," and all other substances used for the purpose of producing insensibility, is, that it is administered by absorption instead of inhalation, and this enables the dose applied to be measured with greater accuracy. On passing into the system it becomes decomposed into formiate of potassium and chloroform, and produces more perfect insensibility than either ordinary chloroform or ether. Its use is said to be unattended by any danger. In a very painful and difficult operation lately performed on a woman M. Liebreich applied chloral with perfect success, the patient being kept under its influence for over two hours.

